# Effect of Health Education on Healthcare-Seeking Behavior of Migrant Workers in China

**DOI:** 10.3390/ijerph17072344

**Published:** 2020-03-30

**Authors:** Xuefeng Li, Han Yang, Hui Wang, Xujun Liu

**Affiliations:** 1 Western China Economic Research Center, Southwestern University of Finance and Economics, Chengdu 611130, China; fzyanghan@126.com; 2 School of Accounting, Southwestern University of Finance and Economics, Chengdu 611130, China; wanghui@swufe.edu.cn; 3 School of Finance, Southwestern University of Finance and Economics, Chengdu 611130, China; lxujun@smail.swufe.edu.cn

**Keywords:** health education, healthcare-seeking behavior, migrant worker, China

## Abstract

Health education is considered to be an effective way to improve the healthcare-seeking behavior of migrant workers. This study examined the impact of health education on healthcare-seeking behavior of migrant workers in China and explored the differences in different health education methods. This paper used the 2017 China Migrants Dynamic Survey (CMDS) to analyze the relationship between health education and healthcare-seeking behavior. Our results indicated that health education could significantly improve the healthcare-seeking behavior of migrant workers, but there was still ample space for improvement. From the perspective of different health education methods, lectures, public consultation, and online education were positively correlated to healthcare-seeking behavior, while publicity materials and bulletin boards were not. Although the effects of publicity materials and bulletins were limited, these two health education methods were still the most widely used. Our results emphasized the necessity of increasing investment in lectures, public consultation, online education, and other similar health education methods. This change in health education methods can play an effective role in the spread of health education to improve the healthcare-seeking behavior of migrant workers.

## 1. Introduction

The term ‘migrant workers’ refers to the labor force groups who work as temporary residents in non-registered locations without changing their household registration [1]. In 2018, China’s migrant workers reached 288.36 million, accounting for 20.7% of the total population [2]. However, due to the restrictions of the household registration system in China, plenty of migrant workers can only live in working cities temporarily, resulting in their unequal treatment by public services [3,4,5]. Among them, the inequality of health services is particularly severe. For example, migration reduces the probability of using health records [6]; most of the migrant workers do not even establish health records in their working city. In order to reduce the health risks caused by inadequate health services and ensure social equity, the Chinese government has formulated a series of policies to improve the availability of health services for migrant workers.

In improving the availability of health services, it is also essential to guide migrant workers to develop scientific healthcare-seeking behavior. To a considerable extent, healthcare-seeking behavior remarkably affects health-related quality of life of migrant workers [7]. Based on an extensive sample survey conducted by the Ministry of Health of China, the utilization of public health services by migrant workers was generally low [8]. Some case studies have also confirmed this conclusion [9,10]. Therefore, it is necessary to analyze the factors that affect the healthcare-seeking behavior of migrant workers. Understanding these factors is essential for health-related measures concerning migrant workers [11]. Among these factors, health literacy is of wide concern. Health literacy refers to the factors that affect a person’s ability to access, understand, and use information about health and health services [12]. Current research proves that health literacy is positively related to healthcare-seeking behavior [13,14,15,16].

People with insufficient health literacy may delay seeking health services due to not knowing preventive measures or the symptoms of the disease [17]. Compared with residents, the health literacy of migrant workers is lower [18]. Especially during the outbreak of large-scale infectious diseases, due to lack of health literacy, some migrant workers do not seek medical treatment or even choose to cross-regional flow, which becomes the carrier of virus transmission. Taking severe acute respiratory syndrome (SARS) as an example, due to their mobility and their lack of health awareness, migrant workers brought difficulties to the government in controlling infectious diseases [19]. COVID-19 broke out during the Chinese Lunar New Year. Most of the migrant workers had returned to their hometown, and rural areas were not the core areas of the epidemic, so the health of migrant workers was less affected. But the current situation does not mean that migrant workers will not be affected or affect others in future epidemics. Therefore, it is of significance to improve the health literacy of migrant workers to ensure that they can actively seek health services after their illness. An improvement in educational literacy not only protects the health service rights of migrant workers but also prevents and controls the spread of diseases.

At present, strengthening health literacy through health education has become the universal suggestion of most scholars to optimize people’s healthcare-seeking behavior [20,21,22]. Research shows that integrating health knowledge into education is a fair and effective way to promote the health of children and adolescents [23]. A study of Indian women found that, after receiving health education, women can significantly improve the utilization of maternal health care [24]. Although several scholars have studied the relationships between health education and healthcare-seeking behavior, few had studied them in migrant workers. There are various methods to disseminate health education. Different health education methods have different knowledge dissemination mechanisms, hence the difference in the corresponding educational effects. The existing research discussed the approaches to health education from various aspects, such as peer group health education, telephone health education, Internet-based training, etc. [25,26]. At present, the most effective method of health education for migrant workers still lacks research.

The purpose of this study is to analyze the relationship between health education and healthcare-seeking behavior of migrant workers in China. We used a representative national survey to explore the following questions: (1) the specific characteristics of healthcare-seeking behavior of migrant workers; (2) whether health education changes the healthcare-seeking behavior of migrant workers; (3) whether existing health education methods show differences in changing healthcare-seeking behavior of migrant workers. Through discussion of the above problems, we put forward some suggestions for improving health education methods.

### Chinese Health Education Background and Literature

Since the 1980s, provincial health education institutions have been set up in various parts of China to undertake the dissemination of health knowledge in the region. Large and medium-sized cities and even some counties have established health-education institutions. Various medical and health institutions at all levels began to establish corresponding health education departments. The communication network of China’s health education system has been gradually developed and improved [27], which provides the primary carrier for health education. To some extent, this means the germination of China’s health education awareness.

In the 1990s, the Chinese government gradually attached importance to the health education of migrant workers. In 1994, China launched the National Health Education Project for 900 Million Farmers (NAHEF), targeting educational objects including migrant workers, marking the official start of health education at the grassroots level in China. With the promotion of NAHEF, health education methods have been continuously improved. The health education of migrant workers has been determined and organized by communities and villages. Based on local culture and customs, information on health education is widely disseminated through mass media. This method combines mass media and interpersonal communication to improve farmers’ health awareness.

In April 2003, NAHEF was renamed as the National Health Promotion Project for Hundreds of Millions of Chinese Farmers (NAHPF). The goal of the project changed from process-oriented health education activities to result-oriented health promotion, marking that the health education of migrant workers has entered a new stage. The Chinese government has further standardized the service forms and requirements of rural health education activities. However, due to the characteristics of sizeable regional mobility and low educational level, the health education effect on migrant workers is not ideal [28,29]. At present, based on the effective use of the existing health education communication network, there is little research on the best method of health education. Therefore, this paper discusses this topic.

## 2. Materials and Methods

### 2.1. Data Source

The data used in this article is derived from the 2017 CMDS. CMDS is an annual, large-scale national survey of the migrant population conducted by the National Health Commission of China. The respondents of this survey are migrants aged 15 and above who are not registered in the sample district (county, city) and have lived in the sample location for more than one month. The sample locations were selected from the areas with a concentrated migrant population in 31 provinces (districts, cities) and Xinjiang Production and Construction Corps, with 32 provincial units in total.

The survey was divided into three sampling stages by a multi-stage probability method proportional to size. The first stage was to select townships (towns and streets) in proportion among 32 provincial units. In the second stage, village committees were selected based on the size of the chosen townships (towns and streets). The third stage was to investigate the qualified individuals from the chosen village committees. The survey comprehensively collected detailed information about the migrant population, including necessary information about public health services, social security, health status, employment, etc. Using diarrhea, fever, skin rash, and common cold as tracer diseases, we selected migrant workers who had suffered from one of these four diseases in the past year as samples. After eliminating some samples with missing data, we obtained 40,951 observations in the final sample.

### 2.2. Measures

#### 2.2.1. Dependent Variables

Healthcare-seeking behavior is a kind of social behavior. People adopt this behavior to confirm the existence of disease and alleviate the pain of disease [30]. We divided the health-seeking behavior into several parts, including attitude to treatment, choice of treatment method, choice of medical institution, medical expenses, etc. At present, China still faces the problem that not enough migrant workers choose to seek healthcare after illness. It is necessary to guide migrant workers to encourage scientific healthcare-seeking behavior. The primary task is forming the right treatment attitude so that they can seek healthcare in time after their illness. Therefore, we chose to seek healthcare after illness as a measure of migrant workers’ healthcare-seeking behavior.

Using diarrhea, fever, skin rash, and common cold as tracer diseases, we constructed the corresponding dependent variables. Specifically, diarrhea indicates whether to seek healthcare when the frequency of diarrhea is more than or equal to three times per day. Fever indicates whether to seek healthcare when the body temperature is higher than 38 ℃. Skin rash indicates whether to seek healthcare when there is abnormal color, bulge, or blister on the skin surface. Common cold indicates whether to seek healthcare when a cold occurs. All of the dependent variables were assigned to 0–1 (1 = seek healthcare after the illness, 0 = otherwise).

#### 2.2.2. Independent Variables

We selected whether migrant workers had received health education (1 = yes, 0 = no) and five methods of receiving health education as independent variables. The methods of receiving health education are lectures (whether to receive health education through health knowledge lectures, 1 = yes, 0 = no), publicity material (whether to receive health education through publicity materials, e.g., newspapers, film and television, 1 = yes, 0 = no), bulletin board (whether to receive health education through bulletin board/electronic display screen, 1 = yes, 0 = no), public consultation (whether to receive health education through public health consultation, 1 = yes, 0 = no), and online education (whether to receive health education through short message service/WeChat/website, 1 = yes, 0 = no).

#### 2.2.3. Control Variables

In order to control other variables that may affect the research results, we used detailed information about the characteristics of each migrant worker as control variables. We controlled gender (1 = male, 0 = female), age, education level, health status, household income, employment status, service project publicity, health record, social security card, and cooperative medical insurance.

More precisely, education level reflects the highest education level of the migrant worker. Responses were given on a 7-point rating scale, ranging from 1–7: 1 (have not attended school), 2 (primary school), 3 (junior high school), 4 (technical school or senior high school), 5 (junior college), 6 (bachelor degree), 7 (master’s degree and above). Health status represents the health level of the migrant worker. Responses were given on a 4-point rating scale: 1 (I don’t have the ability to take care of myself), 2 (unhealthy, but have the ability to take care of myself), 3 (relatively healthy), 4 (healthy). Household income is the logarithm of the average monthly income of the household in the past year. Employment status reflects whether the migrant worker has had paid jobs in the past week (1 = yes, 0 = no). Health service publicity reflects whether the migrant worker knows the national project concerning essential public health services (1 = yes, 0 = no). Health record depends on whether the migrant worker has established a resident health record (1 = yes, 0 = no). Social security card reflects whether the migrant worker has applied for personal social security card (1 = yes, 0 = no). Basic medical insurance indicates whether the migrant worker participated in basic medical insurance (1 = yes, 0 = no).

### 2.3. Estimation Method

The dependent variables in this paper were 0-1 indicator variables. Considering that the Probit model could deal with heteroscedasticity and non-normality better than the linear probability model [31], we used the Probit model to estimate the probability of healthcare-seeking behavior when migrant workers were ill. The regression model was as follows:

(1) $$Y=\alpha +\varepsilon +\underset{i}{\sum }\beta_{i}X_{i}+\underset{j}{\sum }\gamma_{j}M_{j}$$

In this model, Y represented the dependent variable to measure healthcare-seeking behavior, X_i_ denoted the independent variables, including different types of health education, M_j_ represented the control variables, and ε represented the error term. The coefficients β and γ represented the partial effects of independent variables and control variables on prediction probability, respectively. We used STATA 13.1 ((Stata Corporation, College Station, Texas, TX, USA) for model regressions.

## 3. Results

### 3.1. Descriptive Analysis

The descriptive statistics of each variable are shown in Table 1. There are 40,951 migrant workers in the sample. Among them, the number of sampled migrants who had diarrhea, fever, skin rash, or common cold was 23,506, 20,266, 6815, and 35,119, respectively, with 29.3%, 51.6%, 55.2%, and 39.4% choosing to seek healthcare. In general, 67.6% of the migrants received health education. Among them, the proportion of migrant workers receiving health education through publicity materials and bulletin boards was higher, with 83.8% and 72.7%, respectively. However, only a few migrant workers received health education through lectures, public health consultation, and online education, accounting for 37.9%, 41.6%, and 38.6%, respectively.

In terms of characteristics of the sampled migrant workers, the proportion of males and females was relatively balanced (51.2% were male). The average age was 35.8 years, the average educational background was higher than junior high school, and health scored relatively healthy on average, indicating that the sampled migrant workers were a middle-aged and strong labor force. 81.1% of the sampled migrants had paid jobs in the past week, and the average monthly income of the household in the past year was 5802 yuan (According to the survey of National Bureau of Statistics of China in 2017, the average number of people in a household was 3 (including urban and rural households), and the per capita annual disposable income of urban residents was 36,396 yuan. That is to say, the average monthly income of urban households was 9099 yuan, far higher than that of migrant workers.). 73.2% of migrants had basic medical insurance, indicating that the popularization of basic medical insurance was excellent. Besides, 57.8% of the migrant workers had heard about the national basic public health service project, 50.6% had a personal social security card, and only 25.7% had created residents’ health records, showing that the development of health services in China still needs to be improved.

Table 2 shows the reasons whey migrants did not choose healthcare-seeking behavior. Among the four kinds of diseases, the reasons for not seeking health care were similar. More than 50% of the migrant workers in the sample thought they had knowledge of because they had heard of it before. This answer was the primary reason why they chose not to seek healthcare after being ill. The second reason was that they were too busy to seek healthcare, accounting for 22.8–32.5% among the four diseases. The proportions of migrant workers who reported “I think my body can be self-healing,” “I am short of money to seek healthcare,” or “It is too much trouble to go to the hospital” were all less than 10%.

In Table 3, we analyzed the differences in healthcare-seeking behavior between migrant workers who had received relevant health education and those who had not. It was observed that people with health education were more likely to seek healthcare. Taking diarrhea as an example, 30.9% of the migrant workers who had received health education chose to seek healthcare after the illness, while only 26.2% of the migrant workers without health education did. Furthermore, among all the methods of health education, a higher proportion of those who had received health education sought healthcare than those who had not. This phenomenon was similar among the four groups. We also took diarrhea as an example. The proportion of migrant workers who went to seek healthcare after attending lectures was 34.3%, while the proportion who did not attend was 28.9%. By the univariate *t*-test, among all kinds of disease and all methods of receiving health education, there were significant differences between the migrants who had received health education and those who had not (*p* < 0.01). In the next section, we use rigorous econometric regression analysis to analyze the relationship between health education and healthcare-seeking behavior.

### 3.2. Regression Results

Table 4 showed the main results of multivariate analysis. The dependent variable was whether a migrant worker choose to seek healthcare after a tracer disease. For each explanatory variable, we reported the estimated marginal effect. In the regression, province dummies were included to control for potential province fixed effects. The results showed that receiving health education was positively associated with healthcare-seeking behavior. In detail, after receiving health education, the probability of migrant workers seeking healthcare for diarrhea increased by 3.2%. The effect of health education on migrant workers with fever, skin rash, and common cold is similar, at 1.9%, 3.9%, and 2.7%, respectively.

Not all health education methods were positively correlated to healthcare-seeking behavior (Table 5). Among the four different tracer disease samples, the results were highly similar. Lectures, public consultation, and online education were significantly positively correlated with migrant workers’ behavior in seeking healthcare after illness. For example, in the sample with diarrhea, migrant workers who received lectures, public consultation, and online education were 3.1%, 3.0%, and 2.9% more likely to seek healthcare, respectively. However, the healthcare-seeking behavior of these four tracer diseases was not significantly related to publicity materials and bulletin boards. Different health education methods showed consistent significance in the four models, which showed that the results were robust.

## 4. Discussion

We found that the proportion of migrant workers in China who chose healthcare-seeking behavior after illness was relatively low. After experiencing diarrhea, fever, skin rash and common cold, the proportions of migrant workers who chose to seek healthcare were 29.3%, 51.6%, 55.2%, and 39.4%, respectively, far lower than the national average [32]. This conclusion supported the view that health advantages of migrant workers might gradually weaken after they arrive in cities with better medical conditions [33]. In the case of unknown etiology, if migrant workers do not choose medical treatment in time, their health may be seriously affected. Especially for fever, cold, and other infectious diseases, not seeking medical treatment may harm other people.

We also analyzed the reasons why migrant workers did not choose healthcare-seeking behavior. “I have heard of it before, I think I have treatment experience” and “I am too busy to seek healthcare” were the main reasons why most of the migrant workers chose not to seek healthcare after being ill. To some extent, these two reasons showed that the patients overestimated their physical ability and ignored their conditions. It is undeniable that experience-based coping can probably obtain the right medicine, and some diseases can also achieve self-healing by the autoimmune system. However, the causes of illness may be various. The wrong judgment may lead to severe consequences such as the aggravation of conditions, which need to be avoided. In addition, “I am too busy to seek healthcare” may also be due to the low labor contract signing rate of migrant workers. According to the survey of the Chinese government, only 35.1% of migrant workers signed labor contracts in 2016 [34], but in the same year, the labor contract signing rate of Chinese enterprises reached more than 90% [35]. Without the guarantee of labor contracts, migrant workers’ work status is precarious and vulnerable. Some migrant workers even get paid by the day, which means that leaving work to seek healthcare will cause wage losses. Future policies need to improve the signing rate of migrant workers’ labor contracts to ensure stability of employment. Besides, the proportion of migrant workers who think they can recover, have lack of money, or feel that they are being troublesome in seeking medical aid is relatively low. This result showed that the objective restrictions represented by lack of money and lack of medical service availability were not the main reasons to restrict most people’s access to health services. This conclusion conflicted with Peng et al. (2010) [9], indicating that the work of the Chinese government in recent years to protect migrant workers’ access to health services has been effective.

Due to government subsidies, the coverage of public health services for migrant workers has been dramatically improved [6]. However, the proportion of migrant workers who choose medical treatment was still low. Therefore, to promote the healthcare-seeking behavior of migrant workers, we should consider not only the supply side of public health services but also the demand side [36]. Society should guide migrant workers to form scientific medical habits. Overall planning of health service supply and demand is crucial to change the underutilization of health services of migrant workers in China. Our results showed that some migrant workers overestimated their abilities and neglected their conditions. To solve this problem, we should carry out effective health education, letting migrant workers understand the diversity of causes and the severity of the disease, to raise their attention and finally improve their medical behavior.

Based on these statistics, we further empirically explored the relationship between health education and healthcare-seeking behavior of migrant workers in China. Our results showed that health education could significantly promote the healthcare-seeking behavior of migrant workers. The regression results were consistent with the existing literature that health education was firmly related to healthcare-seeking behavior [24], indicating that China’s policy has had some achievements in the implementation of health education for migrant workers. The popularization of health education is an effective way to improve migrant workers’ healthcare-seeking behavior. However, considering the four tracer diseases, the probability that health education could improve migrant workers’ healthcare-seeking behavior was less than 4%, indicating that there was ample space for improving health education. Meanwhile, the proportion of migrant workers who had received health education was less than 70%. The popularity of health education still needs to be improved.

We also found that not all health education methods were positively correlated to healthcare-seeking behavior. Lectures, public consultation, and online education were the health education methods that positively associated with healthcare-seeking behavior. At the same time, publicity material and bulletin boards were not significantly correlated to healthcare-seeking behavior. This result showed that migrant workers were more inclined to face-to-face learning like lectures and public consultation when they received health education. The health education methods of publicity materials and bulletin boards tended to let migrant workers learn by themselves. This traditional way of education might be annoying, making it difficult to stimulate an interest of learning [37,38]. Online education also requires self-study but played a significant role in improving the healthcare-seeking behavior of migrant workers. The reason for this phenomenon may be that online education had sufficient information and more convenient access to information. With the increasing popularity of the Internet, online education with lower cost and more diverse forms will be an essential approach to health education in the future.

It was worth noting that publicity material and bulletin boards, which had no significant impact on the medical behavior of migrant workers, were still the two most commonly used health education approaches of the government. Publicity material and bulletin board accounted for 83.8% and 72.7% of the migrant workers who had received health education, respectively. Lectures, public consultation, and online education, which had significant impacts on migrant workers, only accounted for about 40%. This condition partly explains the results that health education had significant but insufficient effects on healthcare-seeking behavior. The possible reason was that for government clerks, traditional education approaches, such as publicity materials and bulletin boards, were the most commonly used methods and the least costly. Lectures and public consultation had higher requirements for human, material, and financial investment, leading to their low availability. As a new method of health education, online education has no ready-made working standard. It needs grass-roots workers to explore and develop by themselves, and the corresponding learning cost is high. Rationally, when the educational effect is not included in the work assessment, these most commonly used method of health education is the best choice for the government clerks.

There are several limitations to our study. First, we chose migrant workers’ healthcare-seeking behaviors as dependent variables to test the effects of health education. The direct effect of health education should be health knowledge [39,40], to improve the medical behavior of migrant workers. The mediating effect of health knowledge between health education and healthcare-seeking behavior may be tested in future research. Second, when measuring healthcare-seeking behavior, we only chose to seek healthcare after illness. However, other indicators of healthcare-seeking behavior, such as where to seek healthcare, were not included in the analysis. Future research can select more variables to measure the concept of healthcare-seeking behavior according to different aspects of patients’ medical treatment. Third, we only chose the method of receiving health education as the independent variables, without distinguishing possible differences in the quality of education. Future research can test the effect and quality of the education method, providing more detailed suggestions for optimizing health education method.

## 5. Conclusions

This paper studied the healthcare-seeking behavior of migrant workers regarding four diseases, namely diarrhea, fever, skin rash, and common cold. To some extent, the objective limitation (e.g., lack of money or medical service availability) of healthcare-seeking behavior faced by migrant workers has been alleviated. However, due to subjective reasons (e.g., the overestimation of physical ability and neglect of disease) caused by lack of education knowledge, the proportion of migrant workers who chose to go to seek healthcare after disease was still low. Health education could significantly improve the healthcare-seeking behavior of migrant workers, but there is still ample space for improvement. Not all health education methods were significantly correlated to healthcare-seeking behavior. Lectures, public consultation, and online education were positively related to healthcare-seeking behavior, while publicity material and bulletin boards were not. More significantly, although the effects of publicity materials and bulletins were limited, these two health education methods are still the two most widely used methods. Therefore, investment in lectures, public consultation, and online education remains to be strengthened. Increasing investment in these three methods, especially improving the standardized implementation mode of online education, is of significance to promote the development of health education. This study provides evidence for understanding the health education of migrant workers and promotes the development of health education. At the same time, with the worldwide outbreak of COVID-19, people in some countries still do not pay enough attention to health, bringing challenges. We hope that this paper can give some inspiration to governments in other countries to improve people’s attention to health through effective health education.

## Figures and Tables

**Table 1:** Descriptive statistics of variables.

Variable	Mean	Standard Deviation	Observation
Diarrhea	0.293	0.455	23,506
Fever	0.516	0.500	20,266
Skin rash	0.552	0.497	6815
Common cold	0.394	0.489	35,119
Health education	0.676	0.468	40,951
Lecture	0.379	0.485	27,683
Publicity material	0.838	0.369	27,683
Bulletin board	0.727	0.446	27,683
Public consultation	0.416	0.493	27,683
Online education	0.386	0.487	27,683
Gender	0.512	0.500	40,951
Age	35.81	11.26	40,951
Education level	3.492	1.218	40,951
Health status	3.671	0.579	40,951
Household Income	8.666	0.820	40,951
Employment status	0.811	0.391	40,951
Health service publicity	0.578	0.494	40,951
Health record	0.257	0.437	40,951
Social security card	0.506	0.500	40,951
Basic medical insurance	0.732	0.443	40,951

**Table 2:** Reasons for not seeking healthcare.

Reasons	Diarrhea	Fever	Skin Rash	Common Cold
I had it before, I think I have treatment experience.	0.553	0.520	0.500	0.533
I think my body can be self-healing.	0.066	0.064	0.091	0.072
I am too busy to seek healthcare.	0.325	0.256	0.228	0.275
I am short of money to seek healthcare.	0.066	0.075	0.089	0.068
It is too much trouble to go to the hospital.	0.054	0.067	0.087	0.055

Note: This question is multiple-choice, and the response “other causes” is not included in the table.

**Table 3:** Descriptive statistics and healthcare-seeking behavior data.

Variable		Diarrhea	Fever	Skin Rash	Common Cold
Health education	Yes	0.309	0.532	0.572	0.412
	No	0.262	0.48	0.509	0.356
	Difference	0.047 *	0.053 *	0.062 *	0.055 *
Knowledge lecture	Yes	0.343	0.556	0.621	0.439
	No	0.289	0.517	0.542	0.395
	Difference	0.054 *	0.038 *	0.079 *	0.045 *
Publicity material	Yes	0.313	0.537	0.583	0.415
	No	0.288	0.51	0.519	0.392
	Difference	0.026	0.027	0.064 *	0.023 *
Bulletin board	Yes	0.318	0.544	0.58	0.421
	No	0.286	0.501	0.549	0.386
	Difference	0.032 *	0.043 *	0.031	0.035 *
Public consultation	Yes	0.344	0.567	0.623	0.447
	No	0.285	0.507	0.536	0.386
	Difference	0.059 *	0.061 *	0.087 *	0.061 *
Online education	Yes	0.342	0.566	0.621	0.445
	No	0.289	0.511	0.541	0.39
	Difference	0.052 *	0.055 *	0.081 *	0.055 *

Note: * denotes statistically significant difference at the 1% significance level. “Yes” means having received the relevant health education, “No” otherwise.

**Table 4:** Effect of health education on healthcare-seeking behavior (Probit).

Variable	Diarrhea	Fever	Skin Rash	Common Cold
Health education	0.032 ***	0.019 **	0.039 ***	0.027 ***
	(0.007)	(0.008)	(0.014)	(0.014)
Gender	−0.025 ***	−0.012	−0.036 ***	−0.023 ***
	(0.006)	(0.007)	(0.012)	(0.012)
Age	0.001 *	−0.002 ***	−0.001 **	−0.001 ***
	(<0.001)	(<0.001)	(0.001)	(0.001)
Education level	−0.009 ***	0.003	0.015 **	−0.008 ***
	(0.003)	(0.004)	(0.006)	(0.006)
Health status	−0.026 ***	−0.031 ***	−0.007	−0.016 ***
	(0.006)	(0.007)	(0.011)	(0.011)
Household Income	−0.001	0.006	0.009	0.005
	(0.004)	(0.005)	(0.007)	(0.007)
Employment status	0.005	−0.028 ***	−0.023	−0.017 **
	(0.008)	(0.009)	(0.016)	(0.016)
Service project publicity	0.040 ***	0.039 ***	0.041 ***	0.046 ***
	(0.007)	(0.008)	(0.013)	(0.013)
Health record	0.017 **	0.030 ***	0.039 **	0.030 ***
	(0.008)	(0.009)	(0.016)	(0.016)
Social security card	0.007	0.019 **	0.029 **	0.015 ***
	(0.007)	(0.008)	(0.013)	(0.013)
Basic medical insurance	0.017 **	0.014	0.014	0.022 ***
	(0.007)	(0.009)	(0.015)	(0.015)
Province level fixed effects	Yes	Yes	Yes	Yes
Observations	23,506	20,266	6815	35,119

Note: *, **, *** denote statistically significant difference at the 1%, 5% and 10% significance level.

**Table 5:** Effect of different health education methods on healthcare-seeking behavior (Probit).

Variable	Diarrhea	Fever	Skin Rash	Common Cold
Knowledge lecture	0.031 ***	0.018 *	0.039 **	0.021 ***
	(0.008)	(0.010)	(0.016)	(0.007)
Publicity material	0.002	−0.003	0.029	−0.007
	(0.011)	(0.012)	(0.020)	(0.009)
Bulletin board	0.010	0.004	−0.021	0.003
	(0.009)	(0.010)	(0.018)	(0.008)
Public consultation	0.030 ***	0.034 ***	0.056 ***	0.033 ***
	(0.009)	(0.010)	(0.017)	(0.007)
Online education	0.029 ***	0.016 *	0.043 ***	0.023 ***
	(0.008)	(0.009)	(0.016)	(0.007)
Control variables	Yes	Yes	Yes	Yes
Province level fixed effects	Yes	Yes	Yes	Yes
Observations	23,506	20,266	6815	35,119

Note: *, **, *** denote statistically significant difference at the 1%, 5% and 10% significance level.
